# 

**Published:** 1870-10

**Authors:** 


### CONTENTS.

ORIGINAL COMMUNICATIONS.

Are the Mineral Acids Formed in the Mouth? ..... 417
The Saving of Time in Dental Operations, ..... 426
Report upon Histology and Microscopy, ..... 434
Aluminum for Dental Purposes ..... 439
Light *vs* Heavy Gold Foil and Crystal Gold ..... 442
Watt and William's Base, ..... 445
Dental Literature, ..... 447
A Successful way of Capping Nerves, ..... 449

SELECTIONS, ..... 450

CORRESPONDENCE, ..... 453

EDITORIAL.

Dental Jurisprudence, ..... 457
Association, ..... 460

